# Urinary tract infections in a geriatric sub-acute ward-health correlates and atypical presentations

**DOI:** 10.1007/s41999-018-0099-2

**Published:** 2018-08-29

**Authors:** Zyta B. Wojszel, Małgorzata Toczyńska-Silkiewicz

**Affiliations:** 1 Department of Geriatrics, Medical University of Bialystok, Fabryczna str. 27, 15-471 Bialystok, Poland; 2 Department of Geriatrics, Hospital of the Ministry of Interior in Bialystok, Bialystok, Poland; 3 Germedica, Specialist Medical Practice, Bialystok, Poland

**Keywords:** Bacterial urinary tract infections, Correlates, Clinical picture, Older people, Hospitalization

## Abstract

**Purpose:**

Bacterial urinary tract infections (UTIs) are the most frequently occurring infectious diseases in the geriatric population. The aim of the study was to determine the prevalence and clinical features of UTIs in geriatric in-patients and their association with health and functional ability characteristics.

**Methods:**

A prospective cross-sectional cohort study was conducted among patients hospitalized on the geriatric ward. Patients were interviewed, examined, and had their hospital records analyzed. An uncontaminated midstream urine sample was collected and cultured in all of the cases suspected for UTI. Relative risks for UTI were counted and multivariable logistic regression model was built.

**Results:**

246 patients were included, 179 (72.8%) women, 210 (85.4%) 75 + -year-olds. Bacterial UTIs occurred in 18.3% of the patients. The main etiological agent was Escherichia coli (73.3%). The most significant predictors of UTI were recurrent UTI and urinary catheter. The typical clinical UTI symptoms occurred in less than half of the cases (only in 11.1% of cases fever was observed). More often, than in patients without UTIs, they reported symptoms such as delirium (28.9% vs. 18%), tachycardia (11.1% vs. 1.5%) or hypotension (20% vs. 12.1%).

**Conclusions:**

Bacterial UTIs affect about 1/5 of hospitalized geriatric patients. The clinical picture of these infections very often is atypical and it indicates a need for diagnostic vigilance.

## Introduction

Several factors (including co-morbidity, malnutrition, immune senescence and social health-related factors) result in an increased incidence of infections in older adult individuals. These can also be important predictors for worse outcomes [1]. Urinary tract infections (UTIs) are one of the most common bacterial disease in older people [2], and are associated with a high probability of hospitalization especially in case of concomitant chronic diseases [3].

Diagnosing UTIs in older people can be a challenge. The symptoms of co-morbidities and the presence of various disabilities often complicate the assessment of the clinical condition of the older patient and the diagnosis of UTI. The quite frequent problems in communication with a geriatric patient (due to deafness, neurological diseases or cognitive deterioration) make it difficult to conduct a reliable examination and obtain reliable information on the signs and symptoms of UTI. Such patients require a comprehensive geriatric evaluation. Difficulties may also concern obtaining urine for examination, for example in the case of accompanying delirium.

A common problem in everyday clinical practice, however, is still insufficient knowledge of the specificity of the clinical picture of UTI in older people, whose share in the general population of patients is increasing. Not only a proper diagnosis of the UTI with different clinical picture but also its proper treatment (the right choice of an antibiotic, and also not starting antibiotic therapy in asymptomatic bacteriuria—ASB) is often a difficult task.

The aim of the study was to determine the prevalence and specificity of signs and symptoms of urinary tract infections in geriatric in-patients and their association with health and functional ability characteristics.

## Methods

### Setting, inclusion criteria

We prospectively analyzed consecutive patients admitted to the geriatric ward (sub-acute care) of the Hospital of the Ministry of Interior in Bialystok, Poland between 1st June and 31st December 2013. The chart review was filled based on an interview with the patient or his/her guardian and using the patient’s medical records.

The geriatric ward has got 21 beds. According to the requirements of the National Health Fund the team that carries out the care over patients includes physicians (at least part of them with specialization in geriatrics), nurses (preferably with specialization in geriatric nursing and long-term care), physiotherapist, and psychologist. Older people (obligatory of the age of 60 or older) with multimorbidity and accompanying physical disability, as well as with cognitive impairment, are referred to it. The majority of patients are admitted in a planned manner (the average waiting time for admission is 3 months), but some patients are admitted in an accelerated mode, most often transferred from other hospital departments, or after a telephone consultation with a patient’s family doctor confirming such a need. All patients admitted to the department are carefully evaluated for their health condition, and it is combined with a comprehensive geriatric assessment performed by the multidisciplinary team. A review (and modification if needed) of patient’s pharmacotherapy, and the assessment of patient’s rehabilitation potential and the establishment of a rehabilitation and long-term care plans are one of the most important parts of the whole procedure. The mean length of stay at the department is 7 days.

### Study design and methods

We performed the clinical evaluation of patients and bacteriological examination of their urine samples. Patients were interviewed using a structured questionnaire regarding the age, gender, form of residence (community dwelling/long-term care institution), place of residence (urban/rural), self-assessment of health status, co-morbidities (of 14 chronic diseases medically confirmed or self-reported: peripheral artery disease, coronary artery disease, chronic cardiac failure, stroke, diabetes/prediabetes, neoplasm, thyroid gland disease, dementia, Parkinsonism, depression, chronic arthritis, chronic wounds/pressure sores, chronic renal disease, and kidney stones), physical ability of an older person based on comprehensive geriatric assessment scales (Barthel Index, Norton Scale), and list of risk factors for UTI (such as history of recurrent UTI, indwelling Foley catheter, history of antibiotic treatment or hospitalization in the last 12 months), clinical symptoms that may indicate UTI (based on medical documentation and an interview with the patient, it was determined what specific or nonspecific symptoms accompany the confirmed UTI). It contained also parameters of nutritional status (body mass index—BMI, albumin level, and number of lymphocytes in blood), and renal function (glomerular filtration rate—GFR and serum creatinine level). The incidence of delirium was assessed by nurses with the delirium observation screening scale (DOS) as part of the standard comprehensive geriatric procedure adopted at the geriatric ward.

A standard physical examination was conducted and the medical records were analyzed. Blood was drawn on admittance to the geriatric ward. An uncontaminated midstream urine sample was collected by each patient for urinalysis. Pyuria was diagnosed when > 5 white blood cells per high-power field were observed. When due to patient’s signs and symptoms UTI was suspected, urinary culture was performed. The patients were adequately instructed to collect their urine samples and were assisted by nurses in case of psycho-physical disability. Microbiological cultures were carried out by standard laboratory methods and etiological agents of UTI were determined. In a quantitative bacteriological study, the colony-forming units (CFUs) were counted.

### Study parameters

A UTI was defined as the presence of a positive urine culture accompanied by urinary symptoms related to the bladder or kidneys and included the occurrence of 10^5^ CFUs/mL or more of a urinary pathogen in a specimen (or 10^3^ CFUs/mL or more if the urinary sample was catheterized). Only the first episode of the positive urine culture per patient was included in the analysis. The symptoms of UTI were defined as the presence of complaints of dysuria, increased frequency of urination, urgency, and/or abdominal discomfort, and/or presence of fever (> 38.0 °C), flank or low back pain. Hereinafter, UTI refers to both lower and upper UTI. The diagnosis of UTI was established solely by the treating physician.

### Statistical analysis

Data were collected and analyzed using IBM SPSS Version 18 Software suit (SPSS, Chicago, IL, USA), and presented as medians and interquartile range for continuous variables (all of them were not normally distributed) and the number of cases and percentage for categorical variables. To compare differences between the groups, we used χ^2^ test or Fisher’s exact test, as appropriate, for categorical variables. Interval data were compared with the use of the Mann–Whitney test. Relative risks (*RR*s) were calculated to evaluate the potential risk factors that might influence the occurrence of UTIs in geriatric in-patients. It was followed by a multivariable logistic regression including all predictors with a *P* value of *RR* less than 0.1. A *P* value of less than 0.05 was regarded as significant.

## Results

246 patients took part in the study. Most were women (179; 72.8%) and 75 + -year-old people (210; 85.4%). The age structure in both gender groups was similar. All of the participants were community dwelling. Patients living in rural area constituted 29.3% of the group. Participants in both age and gender groups assessed their health status in a similar way—in 63.4% of cases as bad or very bad.

Significant bacteriuria in the urinary culture was found in 53 (21.5%) of patients, but clinical evaluation revealed that only 18.3% (*n* = 45) of cases met the diagnostic criteria for UTI. 3.2% (*n* = 8) of cases was classified as asymptomatic bacteriuria (ASB). The predominant causative pathogen was *Escherichia coli (E. coli),* isolated in 33 (73.3%) of UTI cases. Other pathogens found in urine samples were *Klebsiella pneumoniae* (22.3%), *Klebsiella oxytoca* (2.2%), and *Proteus mirabilis* (2.2%).

To assess the homogeneity of the groups and evaluate the possible risk factors of UTI, we compared patients diagnosed with UTI with those without it (Table 1). The groups did not differ in age, gender, place of residence, the number of chronic diseases, BMI, albumin level, lymphocyte number, GFR and creatinine level. However, the Barthel Index and Norton scale scores were significantly lower in patients with UTI when compared with those without this diagnosis. The white blood cell number and CRP differed between the groups, achieving significantly higher values in patients with UTI.

**Table 1:** Characteristics of the study groups

Parameter	Patients with UTI	Patients without UTI	*P* value^a^
No. (%) of patients	45 (18.3)	241 (81.7%)	
Age, years	84 (77–88)	83 (78–87)	0.41
Gender, female, *n* (%)	34 (75.6)	145 (72.1)	0.64
Place of residence, rural, *n* (%)	59 (29.4)	13 (28.9)	0.99
Barthel Index	77.5 (27.5–90.0)	90 (70–95)	0.003
Norton scale	15 (12–18)	17 (15–18)	0.04
Multimorbidity^b^	6 (5–8)	5 (4–6)	0.78
White blood cells, G/L	7.2 (6.0–9.1)	6.5 (5.4–8.2)	0.02
CRP, mg/L	5.5 (3.2–13.9)	2.7 (1.2–6.5)	< 0.001
BMI, kg/m^2^	28 (26–34)	28 (25–32)	0.32
Albumin, g/L	38 (35–42)	40 (37–42)	0.12
Lymphocytes, K/μL	1.48 (1.18–1.98)	1.57 (1.23–1.99)	0.64
GFR^c^, mL/min/1.73 m^2^	49.9 (39.9–61.0)	49.4 (37.3–60.7)	0.94
Serum creatinine, μmol/L	106.1 (81.3–122.0)	89.3 (75.1–111.4)	0.2

Median values (IQR) are shown unless otherwise indicated

*BMI* body mass index, *CRP* C-reactive protein, *GFR* glomerular filtration rate, *IQR* interquartile range

^a^χ^2^ test or Fisher’s exact test, as appropriate, for categorical variables. Mann–Whitney test for interval variables

^b^The number of chronic diseases of 14 included in the analysis

^c^Estimated using the Cockroft-Gault equation

Our analyses revealed possible risk factors for UTI (Table 2). The most significant factors were recurrent UTI (risk ratio, 10.8; 95% CI 3.4–33.8; *P *< 0.001) and urinary catheter (risk ratio, 4.6; 95% CI 2.8–7.5; *P *< 0.001). Patients with severe physical disability presented a 2.7-fold higher risk of UTI (95% CI 1.6–4.6; *P *= 0.001), and reporting antibiotic treatment in the last 12 months a 2.0-fold higher risk (95% CI 1.2–3.4; *P *= 0.008). The risk of UTI was 2.1-fold higher (95% CI 1.1–3.9; *P *= 0.03) if the chronic wounds were diagnosed and in patients with the pressure sores risk (risk ratio, 2.0; 95% CI 1.2–3.4; *P *= 0.02).

**Table 2:** Clinical characteristics and risk factors for urinary tract infections in the study group

Parameter	Patients with UTI *n* = 45	Patients without UTI *n* = 241	RR (95% CI)	*P* value^a^
	*n* (%)	*n* (%)		
Age ≥ 75 years	39 (86.7)	171 (85.1)	1.1 (0.5–2.4)	0.79
Chronic diseases				
1. Peripheral artery disease	40 (31.1)	42 (20.9)	1.5 (0.9–2.7)	0.14
2. Coronary artery disease	42 (93.3)	167 (83.1)	2.5 (0.8–7.6)	0.08
3. Chronic cardiac failure	38 (84.4)	137 (68.2)	2.2 (1.0–4.7)	0.03
4. Stroke	11 (24.4)	29 (14.4)	1.7 (0.9–3.0)	0.10
5. Diabetes/prediabetes	22 (48.9)	81(40.3)	1.3 (0.8–2.2)	0.29
6. Neoplasm	7 (15.6)	26 (12.9)	1.2 (0.6–2.4)	0.64
7. Thyroid gland disease	6 (13.3)	36 (17.9)	0.7 (0.3–1.7)	0.46
8. Dementia	19 (42.2)	74 (36.8)	1.2 (0.7–2.0)	0.50
9. Parkinsonism	7 (15.6)	35 (17.4)	0.9 (0.4–1.9)	0.76
10. Depression	26 (57.8)	127 (63.2)	0.8 (0.5–1.4)	0.50
11. Chronic arthritis^b^	45 (100.0)	196 (97.5)	–	–
12. Chronic wounds	8 (17.8)	15 (7.5)	2.1 (1.1–3.9)	0.03
13. Chronic renal disease	27 (60.0)	86 (42.8)	1.8 (1.0–3.0)	0.04
14. Kidney stones	5 (11.1)	18 (9.0)	1.2 (0.5–2.8)	0.65
Prostate hypertrophy^c^	12 (26.7)	50 (24.9)	1.1 (0.6–2.0)	0.8
Urinary incontinence^d^	31 (68.9)	117 (58.2)	1.5 (0.8–2.6)	0.19
Fecal incontinence^e^	2 (4.4)	1 (0.5)	3.8 (1.6–8.8)	0.09
Urinary catheter	6 (13.3)	2 (1.0)	4.6 (2.8–7.5)	< 0.001
RUTI^f^	42 (93.3)	97 (48.3)	10.8 (3.4–33.8)	< 0.001
Antibiotic in the last 12 months	23 (51.1)	61 (30.3)	2.0 (1.2–3.4)	0.008
Hospital stay in the last 12 months	18 (40. 0)	62 (30.8)	1.4 (0.8–2.4)	0.24
Albumin < 35 g/L	9 (21.4)	27 (14.3)	1.5 (0.8–2.8)	0.25
Lymphocytes < 1.5 K/μL	24 (53.3)	86 (45.3)	1.3 (0.8–2.2)	0.33
BMI > 30 kg/m^2^	14 (40.0)	54 (31.8)	1.3 (0.7–2.5)	0.35
Severe physical disability^g^	11 (25.0)	15 (7.9)	2.7 (1.6–4.6)	0.001
Pressure sore risk^h^	16 (37.2)	36 (19.3)	2.0 (1.2–3.4)	0.02

*n* number of patients, *BMI* body mass index, *CI* confidence interval, *RR* relative risk

^a^χ^2^ test or Fisher’s exact test, as appropriate, for categorical variables. Mann–Whitney test for interval variables

^b^RR countable only for “not having UTI”− 0.8 (0.8–0.9), *P *= 0.29

^c^Men only, *n* = 67

^d^Defined as any involuntary leakage of urine

^e^Defined as any involuntary leakage of feces

^f^Recurrent UTI defined as ≥ 2 UTIs in 12 months

^g^Defined as ≤ 20 points in Barthel Index

^h^Defined as ≤ 14 points in Norton scale

No significant increase in the risk of UTI was observed for the majority of chronic diseases included in the analysis, while in chronic cardiac failure, the risk was 2.2-fold higher (95% CI 1.0–4.7; *P *= 0.03), and in chronic renal disease 1.8-fold higher (95% CI 1.0–3.0; *P *= 0.04). We found no significant correlation between hospital stay in the last months and the presence of UTI. In addition, decreased albumin level, decreased lymphocytes number and BMI were not associated with the occurrence of UTI.

In multivariable logistic regression analyses, an independent effect associated with the incidence of the UTI was observed among patients with the recurrent UTI (odds ratio, 14.7; 95% CI 4.0–53.8; *P* < 0.001), or with indwelling urinary catheter (odds ratio, 6.5; 95% CI 1.1–37.8; *P* = 0.04 r), when controlling for antibiotic treatment in the previous 12 months, severe physical disability, chronic cardiac failure, chronic renal disease, pressure sore risk, having chronic wounds, and fecal incontinence (Table 3).

**Table 3:** Risk factors associated with urinary tract infection-multivariable logistic regression model

	OR	95% CI	*P* value
Urinary catheter	6.5	1.1–37.8	0.04
Recurrent UTI	14.7	4.0–53,8	< 0.001
Antibiotic in the last 12 months	0.99	0.4–2.3	0.99
Chronic cardiac failure	2.1	0.8–5.8	0.13
Chronic renal disease	0.7	0.3–1.7	0.48
Pressure sores/chronic wounds	1.7	0.5–5.7	0.41
Fecal incontinence	6.9	0.3–167.8	0.24
Pressure sore risk	0.7	0.2–2.1	0.52
Severe physical disability	2.2	0.6–8.9	0.25

The symptomatology typical for urinary tract infections (Table 4) was observed significantly more frequently in patients diagnosed with UTI: increased frequency of urination (62.2% vs. 19.6%; *P *< 0.001), micturition pain (44.4% vs. 4.0%; *P *< 0.001) and urgency (31.1% vs. 1.5%; *P *< 0.001). Bacteria in the urinary sample were observed only in 51.1% of patients with UTI, and pyuria in 62.2% of them. Only 11.1% of patients with UTI had a fever. In this group, more often than in patients without UTI tachycardia and a tendency to more frequent delirium and hypotension were found.

**Table 4:** Clinical signs and symptoms in patients with UTI and without UTI

Symptoms	UTI *n* = 45	No UTI *n* = 241	*P* value^a^
	*N* (%)	*N* (%)	
Bacteria in urinary sample	23 (51.1)	30 (14.9)	< 0.001
Pyuria^b^	28 (62.2)	55 (27.4)	< 0.001
Hematuria^c^	10 (22.2)	32 (15.9)	0.31
Fever^d^	5 (11.1)	9 (4.5)	0.09
Abdominal pain	11 (24.4)	7 (3.5)	< 0.001
Urgency	14 (31.1)	3 (1.5)	< 0.001
Frequency	28 (62.2)	39 (19.6)	< 0.001
Pain when urinating	20 (44.4)	8 (4.0)	< 0.001
Flank pain/renal angle tenderness	9 (20.0)	7 (3.5)	< 0.001
Delirium^e^	13 (28.9)	36 (18.0)	0.1
Tachycardia	5 (11.1)	3 (1.5)	0.006
Hypotension	9 (20.0)	24 (12.1)	0.16
Loss of appetite	4 (8.9)	12 (6.0)	0.48
Faints/falls, *n* (%)	134 (28.9)	59 (29.5)	0.94

^a^χ^2^ test or Fisher’s exact test, as appropriate, for categorical variables

^b^Defined as > 5 leukocytes/μL

^c^Defined as > 5 RBCs/μL

^d^Defined as temperature > 38.0 °C

^e^Diagnosed with the delirium observational scale

## Discussion

The study confirmed that urinary tract infections were a common problem for patients in the geriatric sub-acute ward—the diagnosis of UTI was made in one-fifth of hospitalized patients—and half of the patients participating in the study reported the problem of recurrent UTI. It agrees with findings from the previous research [4, 5], although definitions for symptomatic UTI vary significantly across the literature, making the reported incidence and prevalence of symptomatic UTI in this population variable [6, 7]. In our study, we followed the criteria of UTI developed by the Polish Ministry of Health within the National Antibiotic Protection Program for the years 2011–2015 [8]. These recommendations were based on the guidelines of Infectious Diseases Society of America and United States Public Health Service, and were generally consistent with them. They did not underline the specific approach to UTI in older people, although some differences in the diagnostic and therapeutic rules in this age group were noticed. The diagnostic criterion for pyuria in this document was >* 5* leukocytes/hpf, whereas in other guidelines the value of above 10 leukocytes/hpf was suggested [6]. The study confirmed also other author’s findings that Escherichia coli remained the major micro-organism responsible for UTIs in hospitalized older people—it was cultured in 73.3% of cases [9, 10].

A characteristic feature of older patients is multimorbidity and high prevalence of so-called geriatric syndromes, such as incontinence, dementia, malnutrition, falls, and depression [11, 12]. All these conditions can make older people more prone to urinary tract infections. Particularly often, these problems occur in nursing homes residents, or among patients admitted to the hospital [13–16], which was also confirmed by our study results. Various chronic diseases and conditions that could predispose to urinary tract infections, such as impairment in activities of daily living, cognitive impairment/dementia, diabetes, prostatic hypertrophy in men, urinary incontinence, and chronic use of urinary catheter were very common in the studied group.

However, in contrast to the results of other authors’ research [17] in our study obesity, several chronic diseases and multimorbidity, signs of malnutrition, or urinary incontinence [18] were not found to correlate with the prevalence of UTI. Generally, high prevalence and co-occurence of these problems in this group as the whole can be one of the reasons.

Our results suggested a possible role for physical disability (represented by low scores in Barthel Index and Norton scale) in the development of UTI. The scientific literature suggests that the relationship between physical fitness and UTIs can be a bilateral one—the more serious physical disability, the higher the risk of UTI, but UTI can also negatively affect the functional ability. Low Norton scale scores were independent predictor of urinary tract infections during rehabilitation in the older patients in the study by Shacham et al. [19], and a low value of Barthel index at admission, high multimorbidity index, and transurethral indwelling catheters promoted the development of nosocomial infections (NI) in acute geriatric inpatients in the study by Marzahn et al. [20]. The occurrence of NIs (most frequently—UTI) further worsened the ADL disability in their study. In other study, patients with UTI seemed to be at risk of inferior functional outcomes also in the case of older people after hip fracture [21].

Apart from physical disability chronic cardiac failure, chronic renal disease, antibiotic use in the previous 12 months seemed to play a significant role in the development of UTI in our study. However, after controlling for all of the factors significantly correlated with UTI, only urinary catheter and recurrent UTI turned out to be the main independent predictors of UTI in our study.

Recurrent urinary tract infection was determined as the most significant risk factor for lower urinary tract symptoms also in the study on lower urinary tract infections in women aged 40 years and over [17]. This indicates that in the case of recurrent UTI the investigations must aim not only to verify the diagnosis of that, but also to find any possible cause of recurrence and implement preventive measures aimed at potential risk factors of UTI [22]. They also include the avoidance of bladder catheterization, that is a common problem especially in hospitalized elderly patients and in the oldest old [23, 24].

The results of our analysis on the risk factors for UTI in the geriatric ward patients may suggest, that maybe in case of very old patients with a number of medical problems—multidimensional measures based on different characteristics of health and psychophysical ability could be more useful in predicting the risk of various complications, including urinary tract infections, then single health characteristics. For example, Multidimensional Frailty Score based on preoperative comprehensive geriatric assessment was useful for predicting postoperative complications (including UTIs) and prolonged hospital stay, even in low-risk elderly women who were undergoing cancer surgery [25], and in patients undergoing different urological procedures [26]. But it has to be verified by other research.

Not only uncertainty in the definition of what constitutes a symptomatic UTI but also the wide variety of presentations makes it one of the most challenging infections to accurately diagnose and effectively treat. Among geriatric patients, the clinical presentation of symptomatic urinary tract infection may be similar to symptomatology in the younger population. However, it is not always evident because typical symptoms are often absent [27, 28]. The only symptom reported may be urinary incontinence, appearing as a new symptom, or worsening of the present one. Other, nonspecific symptoms, such as deterioration of the patient’s general condition, dizziness, falls, drowsiness, fatigue and anorexia, may indicate UTI, even in the absence of fever- rarely present in old age (only 11.1% of patients with UTI presented with a fever in our study). Sudden deterioration in functional abilities, whether a sudden change in mental state (for example, lethargy or a delirium syndrome), may constitute a picture of urinary tract infection specific to this population. Infections, mainly lung and UTIs, followed by drugs and hydro-electrolytic disorders seem to be the most frequent precipitating factors for delirium in community-dwelling older individuals [29]. It is a frequent cause of unnecessary, erroneous diagnoses of mental illness or dementia and psychiatric interventions. In our study, 20.0% of geriatric department patients had delirium. This percentage increased to 28.9% in the presence of UTI. These rates were almost as high as were reported for instance by Pereira and Lopez, who observed delirium in 17.9% of hospitalized older people (in 15% of patients without UTI and in 36.8% of patients with UTI, *P* = 0.018) [30]. Furthermore, non-specific symptoms, such as tachycardia or hypotension, were found in the present study more often in patients with UTI than in those without it, which indicates the need for special vigilance in these cases. Classical clinical symptoms of UTI—apart from frequency—occurred in the study group in less than half of the patients. This is concurrent with the observations of other authors [31, 32].

Chronic urinary symptoms, especially in patients with a catheter, do not always prove UTI [33]. In our study, they were observed also in patients without UTI (particularly increased frequency of urination). It should be remembered that in older people with nonspecific ailments from the lower urinary tract, one should always exclude urinary tract tumors first. Recurrent dysuria symptoms, accompanied by pyuria and sterile urine culture, may suggest atypical urinary tract infection (for example tuberculosis) or may be due to non-infectious causes such as calculi. Nocturia can also be the result of decompensate congestive heart failure.

The study has confirmed a high prevalence of bacteria (14.9%), and pyuria (in 27.4%) in urinary samples of patients without UTI. Bacteriuria is more specific and sensitive than pyuria for detecting urinary tract infection, even in older women. Pyuria is commonly found in the absence of infection, particularly in older adults with lower urinary tract symptoms such as incontinence. Positive testing may increase the probability of urinary tract infection, but initiation of treatment should take into account risk of urinary tract infection based on symptoms as well. In cases in which the probability of urinary tract infection is moderate or unclear, urine culture should be performed [7]. The prevalence of asymptomatic bacteriuria (ASB) with the positive urine culture was quite low (3.2%) in our study compared to the results of other researchers [34, 35]. However, the study was conducted under normal clinical work in the geriatric ward. Therefore, the study group was not a randomly chosen sample from the general population, and the diagnostic procedures had to be in accordance with the relevant guidelines. Abnormal urine test result or accompanying chronic clinical symptoms did not always result in bacteriological examination, but urine culture was ordered mainly in the clinically justified suspicion of UTI. As it has been well documented that clinicians should not screen older adults for ASB and ASB should not be treated with antibiotics [36], it was even expected that the frequency of ASB would be lower in these conditions than in studies aimed at assessing its occurrence in different populations. This result may suggest that the institution’s culture in the field of ordering bacteriological urine tests was quite good. Especially taking into account the fact that—according to Polish recommendations for the diagnosis, treatment and prevention of urinary tract infections in adults, based on which we followed in our study [8]—pyuria was defined as > 5 WBC/hpf.

ASB was benign and did not require treatment, according to the international management guidelines [37]. But overtreatment of asymptomatic bacteriuria (ASB) is widespread and may result in antibiotic side-effects, excess costs to the healthcare system, and may potentially trigger antimicrobial resistance. Treating laboratory findings without taking the clinical picture into account is one of the main mechanisms [38].

The study has got some limitations, which should be mentioned. First of all, it was performed not in the sample randomly selected from the general population of older people, so the results can be generalized for the patients of the similar settings only. Besides, UTI symptom such as pyuria was diagnosed based on the criterion that was not used by all authors, which might make comparisons of the study results more difficult. The recently published expert review on UTI across the age groups had confirmed, that pyuria (the presence of leukocytes in microscopic urinalysis) was defined by different researchers as > 5–10 leukocytes/hpf [7], and that, of course, can affect the prevalence of diagnosed ASB or UTI. Some data collected during the study, such as the prevalence of chronic diseases, was partially based on self-reporting, which can be of less accuracy. However, the study was carried out in a hospital setting, and information obtained from the patient was verified by a clinical examination, by an interview with his or her carer, and by a review of all of the patient’s medical records available. And only those chronic conditions that were confirmed in some way, or newly detected during the current hospitalization, were included in the analysis. There is still a risk that some conditions had not been detected/not taken into account, but it could be rather a small percentage in the study group, considering the broad panel of diagnostic tests performed in every case. Other limitations concern the assessment of psycho-physical disability. First of all, the physical disability assessment of patients was limited in our study to Barthel Index and Norton scale. It was because—based on our previous clinical experience—we assumed that only the more severe impairment in daily life activities can be connected with the risk of UTI in older people, but this assumption may not be entirely true. All patients hospitalized in the geriatric ward were assessed for cognitive impairment. However, we decided not to include the information on the level of cognitive deterioration in the analysis, as in some patients it did not necessarily result from the presence of dementia. In case of accompanying depressive disorders or delirium, the diagnosis of dementia was postponed, and the patients were referred to the geriatric out-patient clinic for re-evaluation. Therefore, we included only information on confirmed dementia in our analysis. And finally, antibiotics used during the last year did not necessarily have to be associated only with a urinary tract infection. Nevertheless, this variable was included in some publications on the assessment of risk factors of UTI (especially the recurrent ones), therefore, we also decided to include it in our work.

In summary, we showed that the prevalence of bacterial UTI in patients hospitalized in the geriatric ward was high and affected about 1 in every 5 of them. Escherichia coli remained the major micro-organism responsible for UTIs in hospitalized older people. Severe physical disability, chronic cardiac failure, chronic renal disease, urinary catheter, recurrent UTI, pressure sores risk/the presence of chronic wounds, and antibiotic use in the previous 12 months seemed to play significant role in the development of UTI in our study. However, after controlling for all of the factors significantly correlated with UTI, only having history of UTI, and chronic indwelling urinary catheter turned out to be the independent predictors of UTI in our study. Many co-morbidities, that were linked to developing UTI in older adults in other studies (such as diabetes, dementia, prostatic hypertrophy in men, or urinary incontinence) did not appear to have association in our study group.

Typical clinical symptoms of UTI—apart from urgency—occurred in less than half of the patients with UTI hospitalized in the geriatric ward. There were atypical symptoms, such as delirium, tachycardia or hypotension more frequently observed in this group, which indicates the need for special vigilance in these cases and considering UTI as a potential cause of these clinical signs and symptoms. On the other hand, the study has confirmed a high prevalence of bacteria, and pyuria in urinary samples of patients without UTI, as well as the presence of symptoms typical for UTI (frequency). These results suggest that waiting for laboratory confirmation of UTI in many cases may reduce unnecessary antibiotics.
